# Association between the increased level of high-sensitive CRP (hs CRP) and non-arrhythmic ECG changes and echocardiographic abnormalities in patients with acute coronary syndrome

**DOI:** 10.22088/cjim.14.1.83

**Published:** 2023

**Authors:** Roholla Hemmati, Yousef Mohsenzadeh, Reza Madadi

**Affiliations:** 1 Department of Cardiology, School of Medicine, Ilam University of Medical Sciences, Ilam, Iran; 2 Department of Cardiology, School of Medicine, Zanjan University of Medical Sciences, Zanjan, Iran

**Keywords:** CRP, Acute coronary syndrome, ECG, Myocardial infarction

## Abstract

**Background:**

Abnormality in the cardiovascular system such as left ventricular dysfunction caused increased serum CRP and change in electrocardiography pattern. The present study aimed to understand the association between increased levels of highly sensitive CRP (hs-CRP) and non-arrhythmic ECG changes and electrocardiographic abnormalities in patients with the acute coronary syndrome.

**Methods:**

This study was done on 120 patients diagnosed with acute coronary syndrome and hospitalized at CCU. The patients were classified into two groups, one group with an increased level of hs-CRP and another with a normal hs-CRP level.

**Results:**

The patients with an increased level of hs-CRP showed a significantly higher level of cardiac enzymes also ST-segment elevation myocardial infarction (STEMI) was seen in the group with an increased level of hs-CRP than those with normal serum hs-CRP level, but another diagnosis including unstable angina, non-STEMI, heart failure, and emergency hypertension was similarly observed in both groups. Two groups were assessed in terms of left ventricular ejection fraction (LVEF), left ventricular end-diastolic diameter (LVeDD) the prevalence of valvular heart disease, and wall motion abnormality, also showed that groups with increased hs-CRP level, ST-segment elevation leads more significant differences than a normal group (P=0.001).

**Conclusion:**

Patients with an increased level of hs-CRP can be diagnosed as STEMI but not valuable to suppose as echocardiographic abnormalities such as left ventricular dysfunction or hypertrophy.

C-reactive protein (CRP) is a kind of protein that normally circulates at a non-sensed low level in the serum, however, in some conditions including acute inflammatory processes, tissue injuries, or acute infections, it produces hepatic cells and rising in circulation can be markedly induced (1,2). In this regard, it has been a growing interest in the use of this marker as a sensitive marker for predicting and following the progression of each clinical condition underlined by inflammatory or infectious processes (3-5). As it has recently been revealed, different abnormal cardiac conditions such as coronary atherosclerosis, ventricular hypertrophy, and other ventricular filling abnormalities, heart failure, and even valvular heart diseases have underlying inflammatory etiologies accompanied by increased levels of inflammatory responses and thus CRP may have a major role to predict various types of cardiovascular diseases even in healthy subjects (6,7). 

This hypothesis has been strengthened by recent histological studies discovering activated circulating leucocytes, evidence of the systemic release of thromboxane, and other inflammatory mediators besides the elevated level of CRP in these abnormal cardiovascular conditions (8, 9). Furthermore, the significant association between the elevation level of CRP with reduced left ventricular systolic and diastolic dysfunction as well as the abnormality in long-term left ventricular remodeling has been well recognized (10,11). Because of the presence of a close association between ventricular systolic and diastolic dysfunction and abnormal changes in electrocardiographic and echocardiographic patterns, it has been recently hypothesized that among disorders such as left ventricular dysfunction may increase serum CRP so it can be concluded that there is a relationship between increased serum CRP and abnormal changes in electrocardiography pattern. This study aimed to investigate the relationship between increased levels of highly sensitive CRP (hs-CRP) and non-arrhythmic ECG changes and electrocardiographic abnormalities in patients with acute coronary syndrome.

## Methods

This cross-sectional study was performed on 120 patients finally diagnosed with acute coronary syndrome and eventually hospitalized at the cardiac care unit ward of Mostafa Khomeini Hospital in Ilam in 2011. The diagnosis of acute coronary syndrome is based on clinical manifestation, rising cardiac enzymes, and electrocardiographic ischemic changes, acute coronary syndrome is especially defined by laboratory blood factors such as troponin blood test in the setting of symptoms of angina or an angina equivalent and/or electrocardiographic changes consistent with a new q or evolutionary ST T-wave (12). According to the Canadian Cardiovascular Society Classification (class III), if the patients had a negative troponin blood test accompanied by unstable angina or rest angina, recent worsening angina, this may have occurred within myocardial infarction (13). 

Baseline characteristics of the participants including demographics, medical history, history of medications, and the level of cardiac enzymes were collected by interviewing on admission or reviewing hospital recorded charts. On admission, the level of hs-CRP was measured using a commercial kit in all participants. The patients were classified into two groups; increased level of hs-CRP (> 1 mg/dl) as the case group and normal hs-CRP level (≤ 1 mg/dl) as the control group. The patients in both groups were monitored during CCU hospitalization and any abnormal non-arrhythmic changes in ECGs were recorded in a study checklist. Also, the levels of lipid profiles were measured in all subjects. 

 Results were presented as mean ± standard deviation (SD) for quantitative variables and were summarized by frequency (percentage) for categorical variables. Continuous variables were compared using the t-test or Mann-Whitney U test whenever the data did not appear to have normal distribution or when the assumption of equal variances was violated across the study groups. Categorical variables were on the other hand compared using the chi-square test. For the statistical analysis, the statistical software SPSS Version 20.0 for windows (SPSS Inc., Chicago, IL) was used. P-values of 0.05 or less were considered statistically significant.

Results were presented as mean ± standard deviation (SD) for quantitative variables and were summarized by frequency (percentage) for categorical variables. Continuous variables were compared using the t-test or Mann-Whitney U test whenever the data did not appear to have normal distribution or when the assumption of equal variances was violated across the study groups. Categorical variables were on the other hand compared using the chi-square test. For the statistical analysis, the statistical software SPSS Version 20.0 for windows (SPSS Inc., Chicago, IL) was used. P-values of 0.05 or less were considered statistically significant.

## Results

Among the 120 patients that suffered from the acute coronary syndrome, 46 (38.3%) faced increased levels of hs-CRP. The inclusion criterion is ACS (acute coronary syndrome). There are almost very rare confounding factors that do not confuse the sample size, so we decided to not only enter this syndrome. Exclusion criteria include non-cardiac cases that increase or decrease CRP levels, as well as old ECG findings and patients with previous MI.

Table number 1 showed the characteristics of both groups (normal hs-CRP and increased hs-CRP). The two groups were similar in gender and age distribution, and prevalence of cardiovascular risk factors such as obesity, diabetes, hyperlipidemia, and hypertension. According to laboratory parameters in groups with increased levels of hs-CRP are usually accompanied by an increased level of fasting blood sugar, triglyceride, and liver enzyme in serum. Patients who had increased levels of hs-CRP had significantly increased levels of cardiac enzymes such as lactate dehydrogenase (LDH), keratin kinase MB (CKMB), troponin I, and also N-acetyl cysteine (NAC) when compared with normal serum hs-CRP status (Table 2). Regarding underlying cardiovascular abnormalities, ST-segment myocardial infarction (STEMI) was more variable in patients with an increased level of hs-CRP but other related conditions such as unstable angina, non-STEMI, heart failure, and emergency hypertension were similarly observed in both groups.

Differences in echocardiographic parameters were seen in the group with increased hs-CRP and normal hs-CRP levels but left ventricular ejection fraction (LVEF), left ventricular end-diastolic diameter (LVeDD), the prevalence of heart disease, aortic insufficiency, mitral regurgitation, tricuspid regurgitation as well as ventricular hypertrophy were compared. (Table 2). Regarding common non-arrhythmic ECG abnormalities (Table 3), statistical ST-segment elevation in different leads had more significant differences in those with elevated hs-CRP levels than in the group with normal hs-CRP condition (19.6 % versus 1.4%, P=0.001) while about ST- segment depression, there was no significant difference between the two groups (36.5% versus 41.3%, P=0.598). In the increased hs-CRP group, ST-segment changes occurred in precordial leads at 37.0%, in limb leads at 13.0%, and in both types of leads at 8.7%; while these frequencies in another group were 18.9%, 8.1%, and 10.8%, respectively with no difference (Table 3). The frequency of inverted T-wave in the two groups was 37.0% and 29.7%, respectively with no difference (P=0.411). 

**Table 1:** Comparing baseline characteristics between the study groups

**Item **	**Normal HS-CRP** **Group (n = 74)**	**Increased HS-CRP group (n = 46)**	**P-value**
Male gender	28 (37.8)	24 (52.2)	0.123
Age, year	59.56 ± 12.52	60.89 ± 14.42	0.596
BMI, kg/m2	28.78 ± 4.66	30.72 ± 23.60	0.493
Married status	74 (100)	43 (93.5)	0.054
Urban residence	55 (74.3)	35 (76.1)	0.828
History of smoking	6 (8.1)	2 (4.3)	0.422
History of diabetes	15 (20.3)	16 (34.8)	0.077
History of hypertension	35 (47.3)	22 (47.8)	0.955
Manifestations			
Chest pain	3 (4.1)	0 (0.0)	0.565
Palpitation	1 (1.4)	0 (0.0)	0.889
Dyspnea	1 (1.4)	0 (0.0)	0.889
Medication			
TCA	1 (1.4)	2 (4.3)	0.733
ACE-inhibitor	1 (1.4)	1 (2.2)	0.737
Thiazides	1 (1.4)	1 (2.2)	0.737
Statins	1 (1.4)	1 (2.2)	0.737
Aspirin	4 (5.5)	0 (0.0)	0.442
Beta-blockers	1 (1.4)	2 (4.3)	0.733
Vital signs			
Heart rate, /min	78.50 ± 15.76	78.57 ± 13.50	0.981
SBP, mmHg	119.42 ± 26.65	125.72 ± 27.28	0.215
DBP, mmHg	77.43 ± 13.11	79.17 ± 13.81	0.490
Laboratory markers			
HDL, mg/dl	57.23 ± 10.98	57.24 ± 15.77	0.997
LDL, mg/dl	105.85 ± 45.21	100.50 ± 27.60	0.472
Triglyceride, mg/dl	122.64 ± 56.85	161.91 ± 88.93	0.004
Total cholesterol, mg/dl	162.54 ± 34.01	172.57 ± 52.04	0.250
SGOT, mg/dl	18.53 ± 16.73	80.96 ± 124.17	< 0.001
SGPT, mg/dl	20.00 ± 14.47	37.54 ± 37.27	< 0.001
FBS, mg/dl	126.59 ± 63.10	154.15 ± 85.70	0.046

**Table 2:** Results of cardiac assessment in the study groups

**Item **	**Normal HS-CRP** **Group (n = 74)**	**Increased HS-CRP group (n = 46)**	
Cardiac enzymes and biomarkers			
Mean LDH level	4.44 ± 3.32	7.33 ± 6.26	0.001
Mean CKMB level	31.19 ± 55.69	91.96 ± 118.96	< 0.001
mean troponin I level	1.03 ± 0.26	1.21 ± 0.41	0.012
Mean NAC level	1.42 ± 0.25	6.47 ± 1.05	< 0.001
ACS diagnosis			
Unstable angina	47 (63.5)	20 (43.5)	0.244
Emergency hypertension	1 (1.4)	1 (2.2)	0.737
Heart failure	3 (4.1)	1 (2.2)	0.589
STEMI	1 (1.4)	5 (10.9)	0.039
NSTEMI	2 (2.7)	1 (2.2)	0.860
LV dysfunction	0 (0.0)	1 (2.2)	0.388
Echocardiography indices			
LVEF, %	28.64 ± 24.03	26.50 ± 21.51	0.623
LVeDD	2.11 ± 0.94	2.13 ± 0.75	0.886
AI	9 (12.2)	1 (2.2)	0.087
TR	6 (8.1)	4 (8.7)	0.728
MR	10 (13.5)	5 (10.9)	0.660
RWMA	3 (4.3)	4 (9.8)	0.469
LVH	5 (6.8)	4 (8.7)	0.695

**Table 3:** Cardiac arrhythmias in the two study groups

Item	Normal HS-CRP Group (n = 74)	Increased HS-CRP group (n = 46)	
Type of ST change			
ST-segment elevation	1 (1.4)	9 (19.6)	0.001
ST segment depression	27 (36.5)	19 (41.3)	0.598
Location of ST change			
Precordial leads	14 (18.9)	17 (37.0)	0.097
Limbs leads	6 (8.1)	6 (13.0)	0.430
Both	8 (10.8)	4 (8.7)	0.999
T wave changes			
T wave inversion	22 (29.7)	17 (37.0)	0.411
T wave tall	1 (1.4)	0 (0.0)	0.999

## Discussion

Our study demonstrated a similar abnormal change in echocardiography parameters between the groups with and without serum hs-CRP elevation regarding left ventricular dysfunction, left ventricular hypertrophy, and wall motion abnormality, however, for non-arrhythmic ECG changes, ST-segment elevation indicating STEMI had more significant differences in those in the increased hs-CRP group. On the other hand, an increased level of hs-CRP could be one of the reasons STEMI effectively predicts the occurrence of STEMI, and thus the elevated level of hs-CRP may have a major role in the pathophysiological basis of STEMI. However, the elevation of this biomarker may not have a central role in the occurrence of other non-arrhythmic ECG changes. A similar finding was found in a study by Adler et al. revealed the level of CRP that significantly increased on the second and third day that following the first angina myocardial infarction and ST elevation shown in electrocardiography pattern (14), in contrast to Okinet et al., demonstrated no significant differences between ST depression and CRP level seen (15). Asselbergs et al. studied 8.076 subjects that demonstrated not only ST-segment and T-wave abnormalities correlated with increased CRP levels, but also Q-wave myocardial infarction associated with increased CRP levels after adjusting for standard cardiovascular risk factors (16). In fact, in our study, although an increased level of hs-CRP could effectively predict STEMI, however, we could not reveal an association between the elevated level of this marker and left ventricular dysfunction assessed by echocardiography. On the other hand, an early increased level of hs-CRP may predict early ischemic events such as STEMI; however, the occurrence of left ventricular dysfunction or other echocardiographic events may need more time. So, it has been shown that increasing somewhat of CRP levels in patients with STEMI may be associated with getting worse diastolic function and increased left ventricular pressure, left ventricular systolic function, or cardiac output. Previous studies showed the relationship between early increased CRP level and left ventricular systolic function, future heart failure, and mortality in patients who had a myocardial infarction (17–21). Besides, our study finding on the insignificant association between the elevated level of hs-CRP and echocardiographic indices such as left ventricular dysfunction and hypertrophy may be affected by considering a narrow range of hs-CRP measurements in our study population that ranged less than 10, hence, by considering a wide range of CRP may reveal a positive association between the elevation of hs-CRP level and left ventricular dysfunction and hypertrophy in echocardiography. 

For explaining pathophysiological fundaments of inducing STEMI following the elevation of hs-CRP, it has been revealed that acute coronary occlusion that caused myocardial infarction could be determined by complement-mediated inflammation factors and human CRP, indicating this in both human and animal studies, can be responsible for some of this complement activation (22).

Some other mechanisms revealed for this event include increased phosphatidyl inositol 3-kinas activity, upregulated inducible nitric oxide synthase, keratin cell signal transduction pathway, nuclear factor k-B, and up-regulated angiotensin II type 1 receptor in vascular smooth muscle cells and direct production of nitric oxide by endothelial cells resulted in increased production of endothelial–1 also increase of von Willebrand factor which is known to be accompanied by endothelial dysfunction (23-27). 

In conclusion, increased levels of serum hs-CRP can effectively be related to the occurrence of STEMI in patients with acute coronary syndrome. The present study could not demonstrate the predictive role of this biomarker for abnormal echocardiographic changes including left ventricular hypertrophy or dysfunction as well as abnormal regional wall motion abnormality probably due to considering a narrow range of hs-CRP (less than 10) or employing a small sample size.
